# Chemical Degradation of Androgen Receptor (AR) Using Bicalutamide Analog–Thalidomide PROTACs

**DOI:** 10.3390/molecules26092525

**Published:** 2021-04-26

**Authors:** Ga Yeong Kim, Chae Won Song, Yo-Sep Yang, Na-Rae Lee, Hyung-Seok Yoo, Seung Hwan Son, Soo Jin Lee, Jong Seon Park, Jong Kil Lee, Kyung-Soo Inn, Nam-Jung Kim

**Affiliations:** 1 College of Pharmacy, Kyung Hee University, 26 Kyungheedae-ro, Dongdaemun-gu, Seoul 02447, Korea; gagoyo1234@khu.ac.kr (G.Y.K.); didtmdrl316@khu.ac.kr (Y.-S.Y.); nrl9758@khu.ac.kr (N.-R.L.); yoohs@khu.ac.kr (H.-S.Y.); thstmd09@khu.ac.kr (S.H.S.); eldtn05@naver.com (S.J.L.); airfixdog@naver.com (J.S.P.); jklee3984@khu.ac.kr (J.K.L.); 2 Department of Life and Nanopharmaceutical Sciences, Graduate School, Kyung Hee University, 26 Kyungheedae-ro, Dongdaemun-gu, Seoul 02447, Korea; chae1@khu.ac.kr

**Keywords:** bicalutamide, protein degradation, PROTAC, androgen receptor (AR), prostate cancer

## Abstract

A series of PROTACs (PROteolysis-TArgeting Chimeras) consisting of bicalutamide analogs and thalidomides were designed, synthesized, and biologically evaluated as novel androgen receptor (AR) degraders. In particular, we found that PROTAC compound **13b** could successfully demonstrate a targeted degradation of AR in AR-positive cancer cells and might be a useful chemical probe for the investigation of AR-dependent cancer cells, as well as a potential therapeutic candidate for prostate cancers.

## 1. Introduction

**Figure 1:**
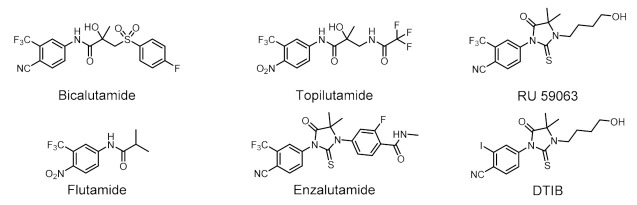
Structures of representative androgen receptor (AR) modulators.

Chemical degraders of specific protein targets, also known as PROTACs (PROteolysis-TArgeting Chimeras), have recently been introduced and considered as part of a novel approach for the treatment of various diseases, especially focusing on pathologies that cannot be alleviated via classical pharmacological therapeutics [1,2,3]. PROTACs are heterodimeric small molecules consisting of a protein target binder, linker, and E3 ligase recruiter. These types of molecules can bind both E3 ligase complexes and target proteins, as well as providing proximity between them. This interaction forms a ternary complex consisting of proteins and enables the target proteins to be ubiquitinated, leading to their proteasomal degradation. In particular, PROTACs require only transient binding to the surface of the target, leading to the catalytic induction of ubiquitination. This catalytic ability of PROTACs enables the target protein to be degraded iteratively and thus address the resistance issues of the existing pharmacological inhibitors, which are generally related to increases or mutations of the target proteins, especially frequently problematic in cancer diseases. Therefore, for the past decade, most of these PROTAC approaches have been developed for targeting refractory cancers including castration-resistant prostate cancer (CRPC) [4,5,6]. In the United States, prostate cancer is one of the most prevalent cancers for men as well as the second leading cause of death by cancer. Once prostate cancer occurs in patients, anti-androgens (also known as androgen receptor (AR) antagonists, which inhibit the signaling mediated by AR that plays a key role in the development of prostate cancer, thus acting as a type of chemical castration) are preferentially prescribed as a first-line therapy for the treatment of these diseases because most prostate cancer cells grow in an androgen-dependent manner [7,8,9] (Figure 1). Alternatively, physical castration to suppress the production of natural androgens can also be carried out for the inhibition of cancer progression. These different types of castration therapies are generally effective in early prostate cancer patients. However, as the treatment time goes by, hormone-dependent prostate cancer becomes progressively worse and is transformed into CRPC, for which the prognosis is very poor. For the treatment of CRPC, next-generation AR antagonists such as enzalutamide have been clinically used [7,8,10,11], but their efficacy is still limited by resistance mechanisms, including increased AR expression and/or AR point mutations for the more potent binding of androgens.

**Figure 2:**
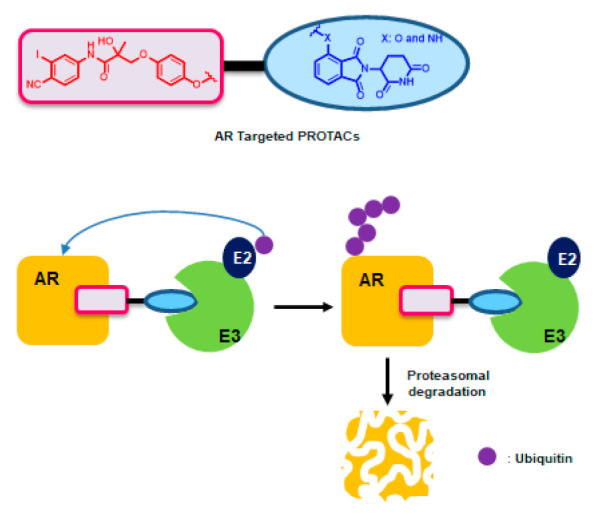
Strategy for androgen receptor (AR) degradation using PROTACs consisting of a bicalutamide analog and thalidomide.

**Scheme 1:**
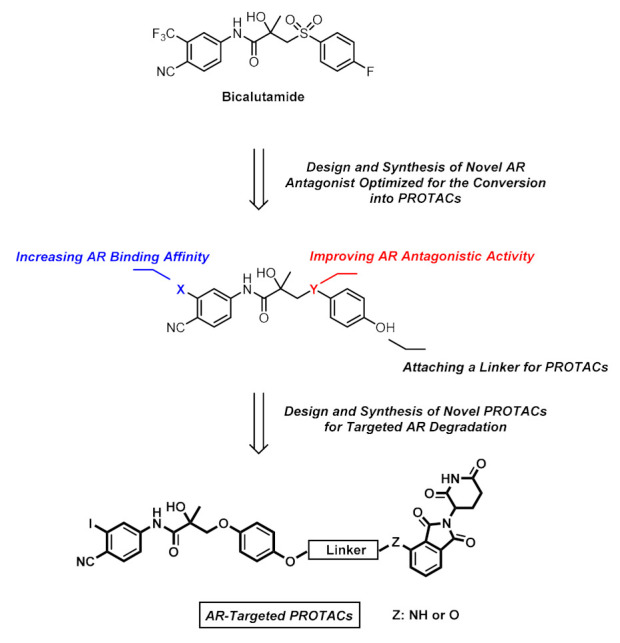
Design strategy for novel AR-targeted PROTACs.

To overcome these unmet medical needs, several PROTACs targeting AR for degradation, not just inhibition, were recently disclosed [4,5,12,13]. Among the AR-targeted PROTACs, ARV-110 was spotlighted and recently entered into clinical trial (Phase 1) in 2019. Given that chemical AR degradation by PROTACs can provide advantages over simple inhibition by existing AR antagonists and that several PROTACs targeting AR have recently been introduced and shown therapeutic potential, we considered it worthwhile to develop novel AR-targeted PROTACs as a potential approach for the treatment of refractory prostate cancers. In this study, we developed a series of novel PROTACs to degrade AR in AR-positive cancer cells. In an effort to successfully develop AR degraders, we preferentially designed and synthesized a series of novel AR antagonists, such as AR ligands (Scheme 1). Upon using a novel AR antagonist as a warhead for AR, we prepared novel PROTACs by conjugating the antagonist to thalidomide, a representative E3 ligase recruiter known as the ligands of cereblon (CRBN) that forms an E3 ubiquitin ligase complex with Cullin-4A (CUL4A) and other compartments, through biologically compatible linking and biologically evaluated them as AR degraders in AR-positive cancer cells (Figure 2).

## 2. Results and Discussion

### 2.1. Synthesis

**Scheme 2:**
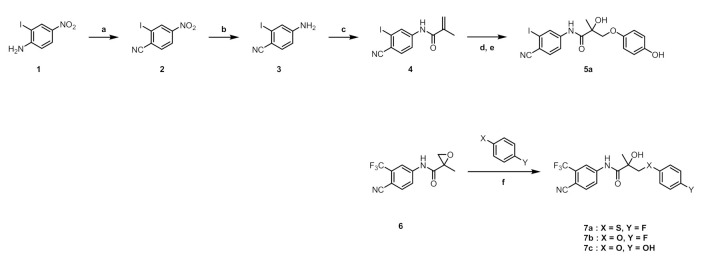
Synthesis of compounds **5a** and **7a**–**c**. Conditions and reagents: (a) NaNO_2_, NaCN, CuCN, H_2_SO_4_, H_2_O, r.t., 50%; (b) SnCl_2_•HCl, EtOH, reflux, 84%; (c) methacryloyl chloride, CH_2_Cl_2_, r.t., 80%; (d) formic acid, hydrogen peroxide, 40 °C; (e) hydroquinone, NaH, DMF, 0 to 90 °C, 55%; (f) NaH, DMF, 0 to 90 °C, 31–95%.

**Scheme 3:**
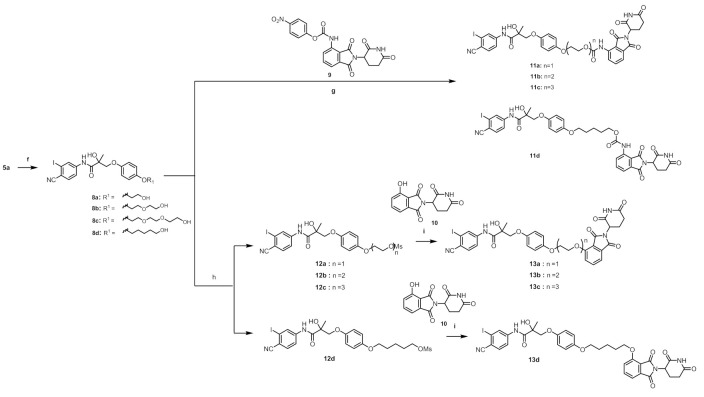
Synthesis of compounds **11a**–**d** and **13a**–**d**. Conditions and reagents: (f) K_2_CO_3_, dry DMF, 90 °C, 54–80%; (g) NEt_3_, CH_2_Cl_2_, r.t., 17–53%; (h) methanesulfonyl chloride, NEt_3_, CH_2_Cl_2_, r.t., 66–81%; (i) K_2_CO_3_, DMF, 90 °C, 27–33%.

To design and synthesize AR-targeting PROTACs, we initially tried to select a nonsteroidal AR antagonist that was clinically more useful than a steroidal AR antagonist in terms of oral availability and off-target side effects as a warhead for targeting AR [14]. Among the various nonsteroidal AR antagonists, bicalutamide, which has been widely used for prostate cancer and acts as an AR antagonist with minor side effects, was selected as an initial template for PROTAC synthesis in our study [10,15]. It was reported that the amide nitrogen and the hydroxyl group of bicalutamide have crucial roles in its anti-androgen activity through hydrogen bonding to the androgen receptor [16,17]. In addition, in 2000, the Van Dort group reported that DTIB where the trifluoromethyl group of RU 59063 was substituted with the hydrophobic iodine had a better binding affinity than RU 59063. Recently, the sulfoxide and fluorine of bicalutamide were reported not to be crucial for the anti-AR activity of bicalutamide [18]. In addition, an attachable moiety of the AR ligand was also necessary to enable it to be conjugated with thalidomide. Collectively, given these previous reports we designed and synthesized a series of AR antagonists such as **5a** and **7a**–**c**, which are expected to provide better AR antagonists as well as a template for PROTAC synthesis (Scheme 2 and Scheme 3). 

Benzonitrile **2** was synthesized from commercially available 2-iodo-4-nitroaniline **1** by Sandmeyer reaction [19]. The nitro reduction of **2** gave 4-aminobenzonitirle **3**, which was followed by acylation with methacryloyl chloride to obtain phenylacrylamide **4** [19,20]. Next, sequential epoxidation and ring opening reactions with hydroquinone were performed to afford **5a** [18,20]. Similarly, from commercially available methacrylamide epoxide bearing trifluoromethyl **6**, a series of thioethers and ethers (**7a**–**c**) were prepared. Based on the preliminary screening results of the AR antagonists that we designed and synthesized, PROTACs **11a**–**d** and **13a**–**d** were synthesized according to the concise and unified synthetic routes described in Scheme 3. PROTACs **8a**–**d** were synthesized by the alkylation of phenol **5a** with alkyl bromide containing several ethylene glycol units [21]. Phthalimide moieties such as **9** and **10** were prepared as shown in the supporting information to be conjugated as ligands for CRBN. The desired PROTAC compounds such as **11a**–**d** were finally obtained by the carbamate coupling of **8a**–**d** with CRBN ligand **9** under basic conditions. To conjugate **8a**–**d** with 4-hydroxythalidomide **10** to synthesize the desired PROTACs **13a**–**d**, primary alcohol moieties in the intermediates **8a**–**d** were converted into the corresponding methanesulfonyl groups [22]. The resultant *O*-mesylates **12a**–**d** were reacted with **10** under the basic conditions to yield other PROTACs such as **13a**–**d**.

### 2.2. Biology

**Figure 3:**
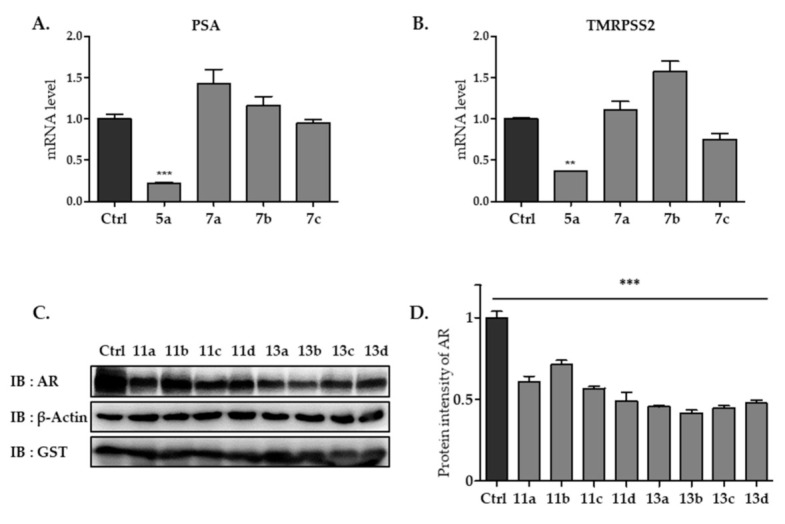
Effects of the novel AR antagonists and novel AR PROTACs. (**A**,**B**) mRNA levels of PSA and TMPRSS2 in LNCaP cells treated with AR antagonists **5a** and **7a**–**c** (10 μM) (n = 3). (**C**) Effects of various AR PROTAC compounds (10 μM) on the levels of ectopically expressed AR in HEK293T cells as determined by immunoblotting. (**D**) Quantitation of protein levels by the treatment of compounds using densitometry. The experiments were repeated at least three times. The data represent mean values ± SEM. *** *p* < 0.001, ** *p* < 0.01. Ctrl, control.

At first, an experiment was conducted to select the most effective AR antagonist among the synthesized antagonists as an AR ligand for PROTAC synthesis. LNCaP cells, one of the AR-dependent prostate cancer cell lines, were treated with **5a** and **7a**–**c** and incubated for 24 h. Additionally, the mRNA levels of AR-dependent transcriptional genes such as prostate-specific antigen (PSA) and transmembrane protease serine subtype 2 (TMPRSS2) in these cells were investigated (Figure 3A,B). As a result, we found that compound **5a** was shown to reduce both the levels of PSA and TMPRSS2 in the most effective manner. Thus, **5a** was found to be more potent than compound **7a,** which was reported to have a slightly better AR antagonistic activity than bicalutamide [18]. This result suggested that the substitution of the trifluoromethyl group and sulfide group with the iodine and ether, respectively, could significantly improve the AR antagonistic activity. In addition, in the case of incorporating the hydroxy group instead of fluorine in the terminal phenyl moiety located at the solvent exposure site, we observed that the AR antagonistic activity was not decreased, indicating that the linker moiety for conjugating the AR ligand with thalidomide could be introduced as an ether linkage on the hydroxy group in that terminal site. With these results in hand, we synthesized a series of AR PROTACs where AR antagonist **5a** was assembled using biocompatible ethylene glycol linkers. Additionally, we evaluated the efficacy of AR degradation by these PROTAC compounds in AR-overexpressing HEK293T cells. Our immunoblotting results indicated that all the compounds could achieve the targeted degradation of AR at the protein level (Figure 3C,D). Among them, compounds **13b** and **13c** reduced the AR protein in highly efficient manners (Figure 3D). Based on these results, we selected compounds **13b** and **13c** as candidate compounds for further study.

**Figure 4:**
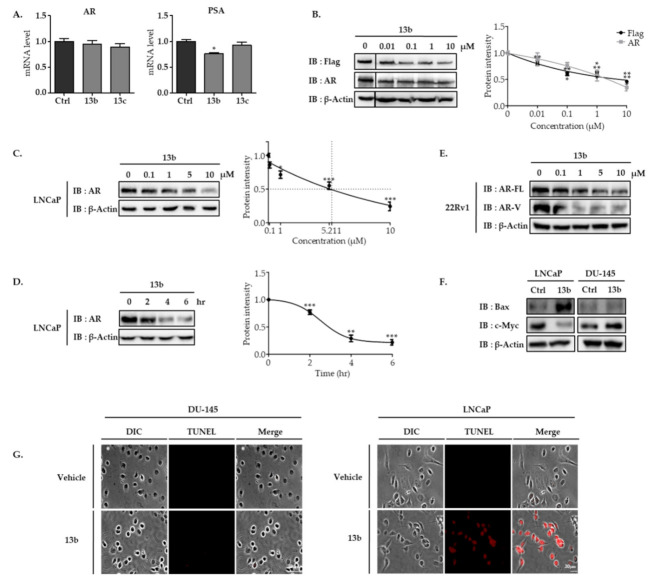
Effects of the novel AR PROTACs. (**A**) mRNA levels of AR and PSA in LNCaP cells treated with AR PROTACs (10 μM) (n = 3). (**B**) Degradation of ectopically expressed AR in HEK293T cells treated with different concentrations of **13b** for 24 h and the quantitative analysis by densitometry. (**C**) Dose-dependent degradation of endogenous AR protein in LNCaP cells treated with **13b** for 24 h. DC_50_: 5.211 μM. (**D**) Time-kinetics of **13b**-mediated degradation of AR in LNCaP cells treated with **13b** (10 μM). All protein intensity analyses were performed in triplicate. (**E**) Effect of **13b** on the protein levels of AR and AR variant (AR-V). 22Rv1 cells were treated with increasing amounts of **13b** for 24 h and the protein levels were analyzed by immunoblotting. (**F**,**G**) AR transcriptional activity-dependent proapoptotic effects of **13b** on prostate cancer cells. LNCaP and DU-145 cells were treated with **13b** for 24 h and subjected to immunoblotting to determine the levels of Bax and c-Myc (**F**). Analysis of apoptosis by TUNEL staining. LNCaP and DU-145 cells were treated with **13b** (5 μM) for 24 h and subjected to TUNEL staining (**G**). The data represent mean values ± SEM. *** *p* < 0.001, ** *p* < 0.01, * *p* < 0.05.

Next, we examined whether the mRNA expression levels of the AR and AR-target genes in LNCaP cells could be affected by the selected PROTACs, such as **13b** and **13c**. As expected, the mRNA levels of AR were rarely altered by the PROTACs. However, compound **13b** suppressed the expression of the PSA gene, while **13c** did not suppress the mRNA level of PSA. In other words, the AR expression was not decreased but the AR-dependent transcription was decreased by PROTAC **13b** at the mRNA level (Figure 4A). To confirm whether **13b** is a targeted AR degrader, a series of immunoblotting analyses was conducted. As shown in Figure 4B, the levels of ectopically expressed AR were decreased by the treatment of compound **13b** in a dose-dependent manner, indicating that the **13b**-mediated decrease in AR is not due to transcriptional regulation via the AR signaling pathway. In a similar manner, AR was shown to be significantly degraded in LNCaP cells after the **13b** treatment in dose-dependent and time-dependent manners (Figure 4C,D). The DC_50_ (degradation concentration 50) value in LNCaP cells was 5.211 μM. The effect of **13b** on AR degradation was further confirmed using 22Rv1, a prostate cancer cell line derived from castration-recurrent xenograft [23]. Interestingly, the AR variant 7 (AR-V) form was much more susceptible to **13b**-mediated degradation (Figure 4E). The AR-V of 22Rv1 is a splicing variant of AR and constitutively active form which enables the prostate tumor to be grown under an environment with insufficient androgen [24]. The result suggested the possible use of the compound as a therapeutic agent to treat castration-resistant prostate cancer through the expression of AR variants. To further analyze the anti-tumor effect of **13b**, the proapoptotic effect of the compound was investigated. Treatment with **13b** resulted in the increment of Bax and the decrement of c-Myc in LNCaP cells, whereas those levels were not significantly affected in androgen receptor-negative DU-145 cells, suggesting that **13b** exerts a proapoptotic effect on AR-dependent cells (Figure 4F). Indeed, robust apoptosis was observed in **13b**-treated LNCaP cells but not in DU-145 cells, as determined by TUNEL staining (Figure 4G). Collectively, our results clearly indicate that PROTAC **13b** enables AR to be targeted and degraded through the direct binding of **13b** with AR, and it does not modulate the mRNA expression level of AR.

### 2.3. Molecular Modeling

**Figure 5:**
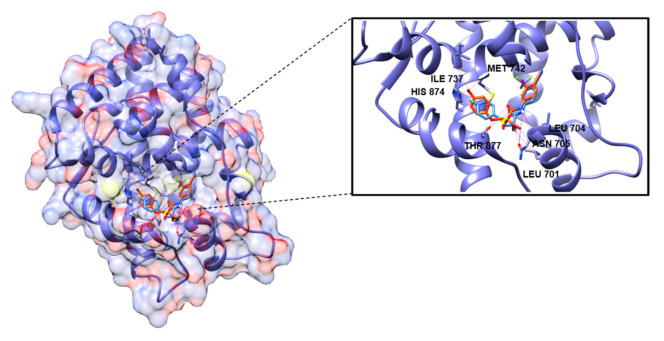
Molecular docking model of **5a** (orange) with the projection of bicalutamide (sky blue) in androgen receptor (PDB code: 1Z95), which was visualized using Chimera 1.10 (UC13bSF Chimera).

To investigate the binding mode of **5a**, which was identified as a novel AR inhibitor in our study, we conducted a molecular docking study of **5a** with AR. Using the X-ray crystal structure of (*R*)-bicalutamide, known as an active enantiomer within AR (PDB code: 1Z95), we performed a molecular modeling of **5a** and observed that the overall binding mode of the compound might be quite similar to that of bicalutamide (Figure 5). The estimated free binding energy of **5a** (−10.71 kcal/mol) was lower than that of **7a** (−9.70 kcal/mol), indicating that **5a** might have an energetically more favored conformation than **7a** (Figure S1). In detail, a tertiary alcohol of **5a**, known as a key pharmacophore for improving the AR antagonistic activity of bicalutamide, forms hydrogen bonds with the amide bonds of Leu701, Asn705, and Thr877, which looks similar to the binding mode of bicalutamide. When fluorine on the terminal phenyl group was replaced with a hydroxyl group, the binding modes of the compounds within AR were likely to be similar [16]. In other words, like fluorine atoms, the phenol group is oriented toward a solvent exposure site, and it seems that phenolic OH can be an optimal site to be conjugated to the linker and thalidomide.

**Figure 6:**
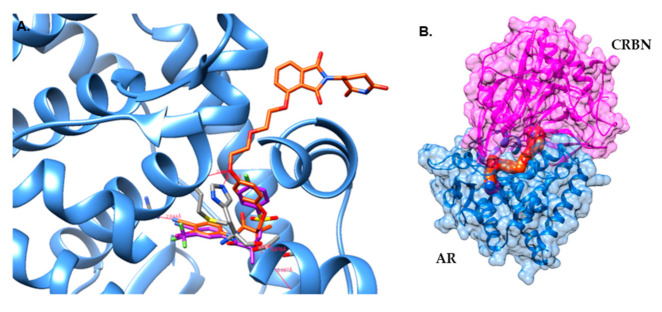
(**A**) Superposition of **13b** (orange) with (*R*)-bicalutamide (purple) bound to androgen receptor (AR, PBD code: 1Z95). The interacting residues are shown in gray. (**B**) Cartoon representation of the ternary complex of **13b** (orange) with AR (PDB Code: 1Z95) and CRBN (PDB Code: 4CI3), which were visualized using Chimera 1.10 (UCSF Chimera).

With the preliminary modeling result of **5a** in hand, to explain the capability of PROTAC **13b** to provide proximity and sequentially induce ubiquitination, we also performed a molecular modeling of **13b** and investigated the feasibility of being assembled into the ternary complex in silico using both the crystal structure of AR bound to (*R*)-bicalutamide and CRBN bound to pomalidomide (Figure 6). In the molecular modeling, AR PROTAC **13b** could be located at the active site of AR and freely extrude the linked thalidomide to the solvent exposure site (Figure 6A). Additionally, it was estimated that the thalidomide region of **13b** could be bound within CRBN (cereblon), appropriately providing proximity between CRBN and AR without mutual steric interactions. For this reason, it was estimated that **13b** might induce the targeted ubiquitination of AR and its resultant degradation at the protein level (Figure 6B).

## 3. Materials and Methods

### 3.1. Chemistry

#### 3.1.1. General Experimental Methods

Unless noted otherwise, all the starting materials and reagents were obtained from commercial suppliers (Aldrich, Acros Organics, Alfa Aesar, and TCI) and were used without further purification. All the solvents used for the routine isolation of products and chromatography were of reagent grade. Reaction flasks were dried at 80 °C. Analytical thin-layer chromatography (TLC) was performed using Merck silica gel glass plates with an F-254 indicator, visualized by UV light (254, 365 nm) and in some cases stained with ninhydrin or *p*-anisaldehyde, followed by heating. Flash column chromatography was performed using silica gel 60 (230–400 mesh) with the indicated solvents. NMR spectra were recorded and obtained using a Bruker 400 (400 MHz for ^1^H NMR) and JMTC 500/54/JJ (126 MHz for ^13^C NMR and 500 MHz for 1H NMR) spectrometer, respectively. 1H and ^13^C were reported in parts per million (ppm) relative to tetramethylsilane, with the residual solvent peak used as an internal reference. Signals are reported as m (multiplet), s (singlet), d (doublet), t (triplet), q (quartet), br (broad signal), and td (triplet of doublets); the coupling constants (*J*) are reported in hertz (Hz). High-resolution mass spectrometry (HRMS) data were obtained with a JEOL JMS-700 instrument (EI-quadrupole). Molecular docking model of 5a (pink) with projection of 7a (sky blue) in androgen receptor (PDB Code: 1Z95), which was visualized using Chimera 1.10 (UC13bSF Chimera) and the synthesis of Pomalidomide and Derivatives are available in Supplementary Materials.

#### 3.1.2. Synthesis

##### 2-Iodo-4-nitrobenzonitrile (**2**)

2-iodo-4-nitroaniline (5.0 g, 18.9 mmol), H_2_SO_4_ (6 mL), and AcOH (22.5 mL) were added to a shading flask at 0 °C. Copper(I) cyanide (2.61 g, 37.8 mmol) in distilled water (4.5 mL) was slowly dropped to the mixture and stirred for 30 min. Then, the mixture was added to copper(I) nitrite (3.39 g, 37.8 mmol), sodium nitrite (1.86 g, 37.8 mmol), and sodium bicarbonate (10.0 g) in distilled water (20 mL). After 1 h, water was added to the flask and extracted with CH_2_Cl_2_. The organic layers were dried with MgSO_4_ and the filtrate was concentrated under reduced pressure. The crude mixture was purified with flash column chromatography to afford compound **2** as a yellow solid (Yield: 61%, 2.09 g). 1H NMR (400 MHz, Chloroform-*d*), δ 8.76 (s, 1H), 8.32 (d, *J* = 8.5 Hz, 1H), 7.82 (d, *J* = 8.5 Hz, 1H). ^13^C NMR (126 MHz, DMSO-*d_6_*), δ 150.09, 136.43, 133.91, 125.42, 124.02, 118.99, 101.89.

##### 4-Amino-2-iodobenzonitrile (**3**)

Tin(II) chloride dihydrate (2.57 g, 11.4 mmol) and compound **2** (626 mg, 2.28 mmol) were dissolved in ethanol (5 mL). The mixture was refluxed for 3 h. Then, the solvent was removed under reduced pressure, followed by extraction with EtOAc and distilled water. The organic layer was dried with Na_2_SO_4_. The filtrate was concentrated under reduced pressure. The crude mixture was purified with flash column chromatography to afford compound **3** as a white solid (Yield: 81%, 445 mg). 1H NMR (400 MHz, Chloroform-*d*), *δ* 7.32 (s, 1H), 7.14 (d, *J* = 2.3 Hz, 2H), 6.63 (d, *J* = 2.3 Hz, 1H), 6.61 (d, *J* = 2.3 Hz, 1H). ^13^C NMR (126 MHz, DMSO-*d*_6_), δ 154.21, 135.71, 123.25, 121.77, 113.50, 103.67, 101.30.

##### *N*-(4-Cyano-3-iodophenyl)methacrylamide (**4**)

Methacryolyl chloride (209.06 µL, 1.8 mmol) was added to a flask and stirred under Ar at 0 °C. Compound **3** (443 mg, 1.8 mmol) was dissolved in DMA dropwise. The mixture was quenched with sodium bicarbonate, followed by extraction with EtOAc and distilled water. The organic layer was dried with MgSO_4_.The filtrate was concentrated under reduced pressure. The crude mixture was purified with flash column chromatography to afford compound **3** as a white solid (Yield: 81%, 430 mg). 1H NMR (500 MHz, DMSO-*d*_6_), δ 10.24 (s, 1H), 8.45 (d, *J* = 2.0 Hz, 1H), 7.91 (dd, *J* = 8.6, 2.0 Hz, 1H), 7.80 (d, *J* = 8.6 Hz, 1H), 5.88 (s, 1H), 5.65 (d, *J* = 1.6 Hz, 1H), 1.96 (s, 3H). ^13^C NMR (126 MHz, DMSO-*d*_6_), δ 167.95, 144.31, 140.25, 135.57, 129.67, 122.08, 119.64, 113.33, 100.77, 19.03.

##### *N*-(4-Cyano-3-iodophenyl)-2-hydroxy-3-(4-hydroxyphenoxy)-2-methylpropanamide (**5a**)

Formic acid (2.3 mL) and 35% hydrogen peroxide (1.4 mL) were dropped in a flask with compound **4** (100 mg, 0.32 mmol). The mixture was stirred for 4 h at 40 °C. After cooling, the reaction was quenched by 2N sodium hydroxide solution. The solution was washed with CH_2_Cl_2_ and dried with MgSO_4._ The filtrate was concentrated under reduced pressure. The crude mixture of *N*-(4-cyano-3-iodophenyl)-2-methyloxirane-2-carboxamide was used for the next step without further purification. Sodium hydride (60% dispersion in mineral oil, 19.2 mg, 0.480 mmol) was added to hydroquinone (70.5 mg, 0.64 mmol) in anhydrous DMF. A solution of *N*-(4-cyano-3-iodophenyl)- 2-methyloxirane-2-carboxamide in anhydrous DMF was slowly added to the reaction mixture and stirred for 1 h at 90 °C. After cooling, the reaction mixture was quenched by 2N HCl solution. The solution was washed with CH_2_Cl_2_ and the organic layer was dried with MgSO_4_. The filtrate was concentrated under reduced pressure. The crude mixture was purified with flash column chromatography to afford compound **5a** as a white solid (Yield: 55%, 70.1 mg). 1H NMR (500 MHz, DMSO-*d*_6_), δ 10.26 (s, 1H), 8.96 (s, 1H), 8.63 (d, *J* = 2.1 Hz, 1H), 8.02 (dd, *J* = 8.6, 2.0 Hz, 1H), 7.81 (d, *J* = 8.6 Hz, 1H), 6.80–6.71 (m, 2H), 6.71–6.63 (m, 2H), 6.19 (s, 1H), 4.15–4.12 (m, 1H), 3.87 (d, *J* = 9.5 Hz, 1H), 1.42 (s, 3H). ^13^C NMR (126 MHz, DMSO-*d_6_*), δ 174.38, 151.42, 151.39, 143.28, 135.02, 129.12, 119.88, 119.20, 115.68, 115.67, 113.03, 100.31, 74.94, 74.33, 23.05. HR-MS (EI) calculated for (M+) 438.0766; found 438.0077.

##### *N*-(4-Cyano-3-(trifluoromethyl)phenyl)-2-hydroxy-3-((4-hydroxyphenyl)thio)-2-methylpropanamide (**7a**)

Sodium hydride (60% dispersion in mineral oil, 60.1 mg, 0.56 mmol) was added to 4-fluorobenzenethiol (78.9 mg, 0.74 mmol) in anhydrous DMF. A solution of *N*-(4-cyano-3-(trifluoromethyl)phenyl)-2-methyloxirane-2-carboxamide (**6**) (101.2 mg, 0.37 mmol) was slowly added to the reaction mixture and stirred for 6 h at 100 °C. After cooling, the reaction mixture was quenched by 2N HCl solution. The solution was washed with CH_2_Cl_2_ and the organic layer was dried with MgSO_4_. The filtrate was concentrated under reduced pressure. The crude mixture was purified with flash column chromatography to afford compound **7a** as a white solid (Yield: 95.7%, 141 mg). 1H NMR (500 MHz, DMSO-*d*_6_), δ 10.51 (s, 1H), 8.45 (d, *J* = 2.0 Hz, 1H), 8.23 (dd, *J* = 8.6, 2.0 Hz, 1H), 8.09 (d, *J* = 8.6 Hz, 1H), 7.44–7.37 (m, 2H), 7.13–6.98 (m, 2H), 3.43 (d, *J* = 13.3 Hz, 1H), 3.36 (s, 1H), 3.26 (d, *J* = 13.3 Hz, 1H), 1.47 (s, 3H). ^13^C NMR (126 MHz, DMSO-*d*_6_), δ 174.84, 161.78, 159.84, 143.14, 136.22, 131.91, 131.89, 131.67, 131.61, 122.73, 117.38, 117.34, 115.87, 115.69, 101.88, 75.22, 44.69, 25.83; HR-MS (EI) calculated for (M+) 398.0712; found 398.0710.

##### *N*-(4-Cyano-3-(trifluoromethyl)phenyl)-3-(4-fluorophenoxy)-2-hydroxy-2-methylpropanamide (**7b**)

Compound **7b** was prepared using a similar method for the synthesis of **7a** (16.7%). 1H NMR (500 MHz, DMSO-*d*_6_), δ 9.18 (s, 1H), 8.11 (d, *J* = 2.1 Hz, 1H), 7.95 (dd, *J* = 8.5, 2.2 Hz, 1H), 7.78 (d, *J* = 8.5 Hz, 1H), 7.01–6.82 (m, 4H), 4.42 (d, *J* = 8.9 Hz, 1H), 3.95 (d, *J* = 8.9 Hz, 1H), 3.56 (s, 1H), 1.58 (s, 3H). ^13^C NMR (126 MHz, DMSO-*d*_6_), δ 174.65, 157.57, 155.69, 154.88, 143.29, 136.38, 122.74, 117.42, 117.38, 116.03, 115.97, 115.88, 115.93, 115.74, 101.93, 74.91, 74.21, 23.03; HR-MS (EI) calculated for (M+) 382.0941; found 382.0939.

##### *N*-(4-Cyano-3-(trifluoromethyl)phenyl)-2-hydroxy-3-(4-hydroxyphenoxy)-2-methylpropanamide (**7c**)

Compound **7c** was prepared using a similar method for the synthesis of **7a** (31.4%). 1H NMR (500 MHz, DMSO-*d*_6_), δ 9.13 (s, 1H), 8.10 (d, *J* = 2.2 Hz, 1H), 7.96 (dd, *J* = 8.5, 2.2 Hz, 1H), 7.81 (d, *J* = 8.5 Hz, 1H), 6.84–6.72 (m, 4H), 4.70 (s, 1H), 4.40 (d, *J* = 9.0 Hz, 1H), 3.92 (d, *J* = 9.1 Hz, 1H), 3.47 (s, 1H), 1.57 (s, 3H). ^13^C NMR (126 MHz, DMSO-*d*_6_), δ 174.79, 151.44, 151.37, 143.33, 136.37, 122.69, 117.39, 117.35, 115.90, 115.68, 101.88, 74.99, 74.34, 23.06; HR-MS (EI) calculated for (M+) 380.0984; found 380.0984.

##### *N*-(4-Cyano-3-(trifluoromethyl)phenyl)-3-((4-fluorophenyl)thio)-2-hydroxy-2-methylpropanamide (**8a**)

A solution of acetone (2 mL) and DMF (0.5 mL) was added to *N*-(4-cyano-3-iodophenyl)-2-hydroxy-3-(4-hydroxyphenoxy)-2-methylpropanamide (**5a**) (190 mg, 0.434 mmol), potassium carbonate (120 mg, 0.868 mmol), and tetrabutylammonium iodide (13.7 mg, 0.043 mmol). The mixture was stirred for 30 min at ambient temperature. 2-Bromoethan-1-ol (0.5 mL) was added to the mixture and refluxed overnight. After cooling, the reaction mixture was quenched by 2N HCl solution. The solution was washed with CH_2_Cl_2_ and the organic layer was dried with MgSO_4._ The filtrate was concentrated under reduced pressure. The crude mixture was purified with flash column chromatography to afford compound **8a** as a yellow oil. (113 mg, 54%). 1H NMR (400 MHz, Chloroform-*d*), δ 8.98 (s, 1H), 8.27 (s, 1H), 7.69 (d, *J* = 5.4 Hz, 1H), 7.55 (dd, *J* = 8.4, 3.4 Hz, 1H), 6.84–6.72 (m, 4H), 5.45 (s, 1H), 4.41–4.30 (m, 1H), 3.96–3.82 (m, 1H), 3.60 (s, 1H), 2.96 (d, *J* = 3.5 Hz, 2H), 2.89 (d, *J* = 3.5 Hz, 2H), 1.55 (s, 3H).

##### *N*-(4-Cyano-3-iodophenyl)-2-hydroxy-3-(4-(2-(2-hydroxyethoxy)ethoxy)phenoxy)-2-methylpropanamide (**8b**)

Compound **8b** was prepared using a similar method for the synthesis of **8a** (56.4%). 1H NMR (400 MHz, DMSO-*d*_6_), δ 10.25 (s, 1H), 8.62 (s, 1H), 8.01 (d, *J* = 8.6 Hz, 1H), 7.79 (d, *J* = 8.6 Hz, 1H), 6.85 (s, 4H), 6.20 (s, 1H), 4.64 (s, 1H), 4.15 (d, *J* = 9.5 Hz, 1H), 4.02 (t, *J* = 4.4 Hz, 2H), 3.91 (d, *J* = 9.5 Hz, 1H), 3.74–3.67 (m, 2H), 3.52 (d, *J* = 3.8 Hz, 2H), 3.49 (d, *J* = 4.0 Hz, 2H), 1.42 (s, 3H).

##### *N*-(4-Cyano-3-iodophenyl)-2-hydroxy-3-(4-(2-(2-(2-hydroxyethoxy)ethoxy)ethoxy)phenoxy)-2-methylpropanamide (**8c**)

Compound **8c** was prepared using a similar method for the synthesis of **8a** (54.8%). 1H NMR (400 MHz, DMSO-*d*_6_), δ 10.23 (s, 1H), 8.60 (s, 1H), 8.00 (m, 1H), 7.78 (dd, *J* = 8.6, 0.9 Hz, 1H), 6.83 (s, 4H), 6.18 (s, 1H), 4.59 (t, *J* = 5.7 Hz, 1H), 4.13 (d, *J* = 10.4 Hz, 1H), 4.02–3.96 (m, 2H), 3.89 (d, *J* = 10.7 Hz, 1H), 3.73–3.65 (m, 2H), 3.58–3.52 (m, 4H), 3.48 (t, *J* = 5.1 Hz, 2H), 3.42 (t, *J* = 5.5 Hz, 2H) 1.40 (s, 3H).

##### *N*-(4-Cyano-3-iodophenyl)-2-hydroxy-3-(4-((5-hydroxypentyl)oxy)phenoxy)-2-methylpropanamide (**8d**)

Compound **8d** was prepared using a similar method for the synthesis of **8a** (80%). 1H NMR (400 MHz, Chloroform-*d*), δ 8.98 (s, 1H), 8.27 (s, 1H), 7.72–7.67 (m, 1H), 7.54 (d, *J* = 8.5 Hz, 1H), 6.75 (s, 4H), 5.42 (s, 1H), 5.10 (s, 1H), 4.36 (d, *J* = 9.0 Hz, 1H), 3.89 (t, *J* = 7.2 Hz, 2H), 3.69 (t, *J* = 6.4 Hz, 2H), 2.92 (d, *J* = 29.3 Hz, 1H), 1.81–1.75 (m, 2H), 1.68–1.59 (m, 4H), 1.54 (s, 3H).

##### 2-(4-(3-((4-Cyano-3-iodophenyl)amino)-2-hydroxy-2-methyl-3-oxopropoxy)phenoxy)ethyl (2-(2,6-dioxopiperidin-3-yl)-1,3-dioxoisoindolin-4-yl)carbamate (**11a**)

Compound **8a** (25 mg, 0.048 mmol), compound **9** (19 mg, 0.438 mmol), and NEt_3_ (6 μL, 0.043 mmol) in DMF were added to a flask and stirred under Ar at room temperature. After 1 h, the mixture was quenched with ammonium chloride, followed by extraction with EtOAc and distilled water. The organic layer was dried with MgSO_4_ and the filtrate was concentrated under reduced pressure. The crude mixture was purified with flash column chromatography to afford compound **11a** as a white solid (20 mg, 53%). 1H NMR (500 MHz, Chloroform-*d*), δ 8.95 (d, *J* = 2.7 Hz, 1H), 8.77 (s, 1H), 8.51 (d, *J* = 8.3 Hz, 1H), 8.31–8.21 (m, 2H), 7.74–7.64 (m, 2H), 7.53 (dd, *J* = 8.5, 4.3 Hz, 1H), 7.48 (ddd, *J* = 20.2, 7.3, 0.7 Hz, 1H), 6.89 –6.76 (m, 4H), 5.29 (s, 1H), 4.94 (ddd, *J* = 12.5, 6.0, 3.0 Hz, 1H), 4.63–4.42 (m, 2H), 4.24–4.15 (m, 3H), 3.87 (dd, *J* = 9.1, 7.9 Hz, 1H), 3.68 (s, 1H), 2.96–2.85 (m, 1H), 2.84–2.68 (m, 2H), 2.19–2.11 (m, 1H), 1.53 (s, 3H). ^13^C NMR (126 MHz, Chloroform-*d*), δ 172.68, 170.74, 168.71, 168.17, 166.82, 153.54, 152.71, 152.33, 141.57, 137.82, 136.46, 134.82, 131.39, 129.48, 123.74, 119.43, 118.78, 117.84, 116.17, 116.00, 115.97, 115.91, 115.66, 114.89, 99.00, 75.69, 73.03, 66.75, 64.62, 49.22, 31.36, 22.98, 22.75; HR-MS (EI) calculated for (M+) 781.0881; found 781.0887.

##### 2-(2-(4-(3-((4-Cyano-3-iodophenyl)amino)-2-hydroxy-2-methyl-3-oxopropoxy)phenoxy)ethoxy)ethyl (2-(2,6-dioxopiperidin-3-yl)-1,3-dioxoisoindolin-4-yl)carbamate (**11b**)

Compound **11b** was prepared using a similar method for the synthesis of **11a** (43%). 1H NMR (500 MHz, Chloroform-*d*), δ 8.98 (d, *J* = 7.6 Hz, 1H), 8.77 (d, *J* = 14.3 Hz, 1H), 8.51 (d, *J* = 8.6 Hz, 1H), 8.29 (d, *J* = 2.1 Hz, 1H), 8.23 (d, *J* = 14.8 Hz, 1H), 7.74–7.63 (m, 2H), 7.54 (d, *J* = 8.5 Hz, 1H), 7.45 (ddd, *J* = 7.3, 2.4, 0.7 Hz, 1H), 6.87 –6.71 (m, 4H), 5.29 (s, 1H), 4.93 (ddd, *J* = 12.7, 5.5, 3.5 Hz, 1H), 4.45–4.28 (m, 2H), 4.07 (ddd, *J* = 5.4, 3.9, 2.7 Hz, 2H), 3.84 (dtd, *J* = 6.0, 4.4, 1.7 Hz, 5H), 2.94–2.85 (m, 1H), 2.83–2.68 (m, 2H), 2.16 (s, 1H), 2.19–2.11 (m, 1H), 1.52 (d, *J* = 1.5 Hz, 3H). ^13^C NMR (126 MHz, Chloroform-*d*) δ 172.76, 170.81, 168.66, 168.05, 166.83, 153.85, 152.88, 152.00, 141.61, 137.91, 136.37, 134.81, 131.33, 129.50, 123.85, 119.45, 118.80, 117.74, 116.10, 115.95, 115.82, 115.76, 115.65, 114.87, 98.99, 73.45, 73.22, 69.74, 69.30, 68.17, 65.01, 49.21, 31.38, 22.98, 22.70; HR-MS (EI) calculated for (M+) 825.1143; found 825.1143.

##### 2-(2-(2-(4-(3-((4-Cyano-3-iodophenyl)amino)-2-hydroxy-2-methyl-3-oxopropoxy)phenoxy)ethoxy)ethoxy)ethyl (2-(2,6-dioxopiperidin-3-yl)-1,3-dioxoisoindolin-4-yl)carbamate (**11c**)

Compound **11c** was prepared using a similar method for the synthesis of **11a** (27%). 1H NMR (500 MHz, Chloroform-*d*), δ 8.97 (s, 1H), 8.84 (d, *J* = 5.6 Hz, 1H), 8.53 (d, *J* = 8.7 Hz, 1H), 8.28 (d, *J* = 1.8 Hz, 1H), 8.16–8.07 (m, 1H), 7.73–7.64 (m, 2H), 7.54 (dd, *J* = 8.5, 2.4 Hz, 1H), 7.47 (d, *J* = 7.4 Hz, 1H), 6.79 (q, *J* = 9.6 Hz, 4H), 5.29 (s, 1H), 4.85 (ddd, *J* = 12.5, 5.3, 2.4 Hz, 1H), 4.40–4.31 (m, 3H), 4.07 (dd, *J* = 5.8, 4.1 Hz, 2H), 3.91–3.82 (m, 3H), 3.78–3.68 (m, 5H), 3.69 (d, *J* = 3.5 Hz, 1H), 3.65 (d, *J* = 3.5 Hz, 1H), 2.91–2.84 (m, 1H), 2.82–2.64 (m, 2H), 2.14–2.07 (m, 1H), 1.60 (s, 3H). ^13^C NMR (126 MHz, Chloroform-*d*), δ 172.99, 170.89, 168.73, 168.08, 166.79, 153.96, 152.97, 151.94, 141.61, 137.97, 136.45, 134.85, 131.37, 129.52, 123.83, 119.48, 118.82, 117.86, 115.91, 115.73, 99.02, 75.87, 73.26, 70.88, 70.80, 69.96, 69.15, 68.08, 65.05, 49.24, 31.04, 23.02, 22.65; HR-MS (EI) calculated for (M+) 869.1405; found 869.1403.

##### 5-(4-(3-((4-Cyano-3-iodophenyl)amino)-2-hydroxy-2-methyl-3-oxopropoxy)phenoxy)pentyl (2-(2,6-dioxopiperidin-3-yl)-1,3-dioxoisoindolin-4-yl)carbamate (**11d**)

Compound **11d** was prepared using a similar method for the synthesis of **11a** (17%). 1H NMR (500 MHz, Chloroform-*d*), δ 8.95 (d, *J* = 4.1 Hz, 1H), 8.80 (d, *J* = 2.7 Hz, 1H), 8.54 (d, *J* = 8.5 Hz, 1H), 8.27 (dd, *J* = 2.1, 1.1 Hz, 1H), 8.13 (d, *J* = 5.9 Hz, 1H), 7.74–7.66 (m, 2H), 7.54 (d, *J* = 8.5 Hz, 1H), 7.49 (d, *J* = 7.2 Hz, 1H), 6.80 (m, 4H), 4.93 (dd, *J* = 12.2, 5.4 Hz, 1H), 4.36 (dd, *J* = 9.0, 4.0 Hz, 1H), 4.23 (tt, *J* = 6.6, 3.4 Hz, 1H), 3.94–3.86 (m, 3H), 3.53 (d, *J* = 6.6 Hz, 1H), 2.96–2.86 (m, 1H), 2.85–2.69 (m, 2H), 2.19–2.11 (m, 1H), 1.84–1.71 (m, 4H), 1.64–1.56 (m, 2H), 1.57 (m, 3H). ^13^C NMR (126 MHz, Chloroform-*d*), δ 172.76, 170.73, 168.89, 167.91, 166.77, 154.20, 153.14, 151.76, 141.55, 138.15, 136.44, 134.82, 131.34, 129.49, 123.82, 119.43, 118.78, 117.76, 116.05, 116.02, 115.69, 115.57, 115.55, 114.83, 98.99, 75.80, 73.26, 68.17, 65.89, 49.25, 31.40, 28.87, 28.47, 22.99, 22.72, 22.47; HR-MS (EI) calculated for (M+) 823.1350; found 823.1347.

##### 2-(4-(3-((4-Cyano-3-iodophenyl)amino)-2-hydroxy-2-methyl-3-oxopropoxy)phenoxy)ethyl methanesulfonate (**12a**)

Mathanesulfonyl chloride (18.7 μL, 0.242 mmol) dropwise added to compound **8a** (77.8 mg, 0.161 mmol) and anhydrous CH_2_Cl_2_ (3 mL) in an ice bath. After 1 h, the solvent was evaporated in vacuo and the crude mixture was purified with flash column chromatography to afford compound **12a** as a white solid (59.6 mg, 66%). ^1^H NMR (400 MHz, Chloroform-*d*), δ 8.95 (s, 1H), 8.29 (s, 1H), 7.70 (d, *J* = 8.0 Hz, 1H), 7.567 (t, *J* = 6.8 Hz, 1H), 6.85 (m, 4H), 4.55 (t, *J* = 4.2 Hz, 2H), 4.19 (t, *J* = 4.0 Hz, 2H), 3.45 (brs, 1H), 3.23–3.21 (m, 1H), 3.11–3.08 (m, 5H), 1.56 (s, 3H).

##### 2-(2-(4-(3-((4-Cyano-3-iodophenyl)amino)-2-hydroxy-2-methyl-3-oxopropoxy)phenoxy)ethoxy)ethyl methanesulfonate (**12b)**

Compound **12b** was prepared using a similar method for the synthesis of **12a** (81%). 1H NMR (400 MHz, DMSO-*d*_6_), δ 10.28 (s, 1H), 8.39 (d, *J* = 1.9 Hz, 1H), 7.92 (dd, *J* = 8.6, 2.0 Hz, 1H), 7.82 (d, *J* = 8.6 Hz, 1H), 6.96–6.83 (m, 4H), 4.45–4.36 (m, 2H), 4.35–4.29 (m, 2H), 4.06–4.00 (m, 2H), 3.79–3.63 (m, 5H), 3.18 (s, 3H), 1.86 (s, 3H).

##### 2-(2-(2-(4-(3-((4-Cyano-3-iodophenyl)amino)-2-hydroxy-2-methyl-3-oxopropoxy)phenoxy)ethoxy)ethoxy)ethyl methanesulfonate (**12c**)

Compound **12c** was prepared using a similar method for the synthesis of **12a** (74%). 1H NMR (400 MHz, DMSO-*d*_6_), δ 10.31 (s, 1H), 8.41 (s, 1H), 7.93 (dd, *J* = 8.7, 2.0 Hz, 1H), 7.84 (d, *J* = 8.6 Hz, 1H), 6.96–6.86 (m, 4H), 4.45–4.38 (m, 2H), 4.35–4. 30 (m, 2H), 4.07–4.01 (m, 3H), 3.75–3.71 (m, 2H), 3.71–3.66 (m, 2H), 3.60 (s, 4H), 3.19 (s, 3H), 1.87 (s, 3H).

##### 5-(4-(3-((4-Cyano-3-iodophenyl)amino)-2-hydroxy-2-methyl-3-oxopropoxy)phenoxy)pentyl methanesulfonate (**12d**)

Compound **12d** was prepared using a similar method for the synthesis of **12a** (67%). 1H NMR (400 MHz, DMSO-*d*_6_), δ 10.30 (s, 1H), 8.41 (d, *J* = 1.9 Hz, 1H), 7.93 (dd, *J* = 8.6, 1.9 Hz, 1H), 7.84 (d, *J* = 8.6 Hz, 1H), 6.94–6.84 (m, 4H), 4.45–4.37 (m, 2H), 4.23 (d, *J* = 12.8 Hz, 2H), 4.05 (q, *J* = 7.1 Hz, 1H), 3.91 (t, *J* = 6.4 Hz, 2H), 1.87 (s, 3H), 1.75–1.71 (m, 2H), 1.55–1.45 (m, 2H).

##### *N*-(4-Cyano-3-iodophenyl)-3-(4-(2-((2-(2,6-dioxopiperidin-3-yl)-1,3-dioxoisoindolin-4-yl)oxy)ethoxy)phenoxy)-2-hydroxy-2-methylpropanamide (**13a**)

Compound **10** (9.9 mg, 0.036 mmol), potassium carbonate (12.4 mg, 0.068 mmol), and anhydrous DMF (0.8 mL) were added to a flask at 90 °C. After 30 min, compound **12a** (25.4 mg, 0.453 mmol) was added to the mixture and stirred for 3 h. The mixture was diluted with H_2_O, followed by extraction with EtOAc and distilled water. The organic layer was dried with MgSO_4_, and the filtrate was concentrated under reduced pressure. The crude mixture was purified with flash column chromatography to afford compound **13a** as a white solid (9.0 mg, 27%). 1H NMR (500 MHz, Chloroform-*d*), δ 8.96 (d, *J* = 2.3 Hz, 1H), 8.27 (dd, *J* = 2.8, 2.1 Hz, 1H), 8.22–8.14 (m, 1H), 7.72–7.62 (m, 2H), 7.53 (dd, *J* = 8.5, 2.2 Hz, 1H), 7.48 (dt, *J* = 7.3, 0.7 Hz, 1H), 7.30 (ddd, *J* = 8.6, 3.9, 0.7 Hz, 1H), 6.92–6.85 (m, 2H), 6.88–6.76 (m, 2H), 5.29 (s, 1H), 4.97–4.89 (m, 1H), 4.52 (td, *J* = 4.5, 2.2 Hz, 2H), 4.41–4.31 (m, 2H), 3.94–3.84 (m, 1H), 3.54 (d, *J* = 9.3 Hz, 1H), 2.91–2.84 (m, 1H), 2.84–2.66 (m, 2H), 2.60 (s, 1H), 1.53 (d, *J* = 1.6 Hz, 3H). ^13^C NMR (126 MHz, Chloroform-*d*), δ 172.79, 170.95, 168.19, 166.94, 165.60, 156.22, 153.56, 152.34, 136.58, 134.81, 133.85, 129.50, 119.66, 118.81, 117.54, 116.53, 116.17, 116.13, 116.09, 116.03, 98.98, 75.81, 73.19, 68.52, 67.25, 49.14, 31.40, 23.00, 22.64; HR-MS (EI) calculated for (M+) 738.0823; found 738.0830.

##### *N*-(4-Cyano-3-iodophenyl)-3-(4-(2-(2-((2-(2,6-dioxopiperidin-3-yl)-1,3-dioxoisoindolin-4-yl)oxy)ethoxy)ethoxy)phenoxy)-2-hydroxy-2-methylpropanamide (**13b**)

Compound **13b** was prepared using a similar method for the synthesis of **13a** (33%). 1H NMR (500 MHz, Chloroform-*d*), δ 8.98 (d, *J* = 6.4 Hz, 1H), 8.28 (dt, *J* = 4.3, 2.2 Hz, 1H), 8.15 (s, 1H), 7.73–7.59 (m, 2H), 7.54 (dd, *J* = 8.5, 0.9 Hz, 1H), 7.48–7.39 (m, 1H), 7.25–7.22 (m, 1H), 6.88–6.74 (m, 4H), 4.91 (dd, *J* = 12.2, 5.5 Hz, 1H), 4.42–4.30 (m, 2H), 4.20–4.02 (m, 4H), 4.02–3.93 (m, 2H), 3.89 (s, 1H), 3.71–3.57 (m, 1H), 2.90–2.83 (m, 1H), 2.82–2.65 (m, 2H), 2.15–2.06 (m, 1H)1.53 (s, 3H). ^13^C NMR (126 MHz, Chloroform-*d*), δ 172.82, 171.09, 168.12, 167.07, 165.54, 156.39, 153.85, 151.98, 136.55, 134.82, 133.74, 129.50, 119.37, 118.81, 117.28, 116.22, 115.98, 115.93, 98.98, 75.83, 73.36, 70.38, 69.56, 69.45, 68.30, 49.10, 29.75, 22.99, 22.61; HR-MS (EI) calculated for (M+) 782.1085; found 782.1087.

##### *N*-(4-Cyano-3-iodophenyl)-3-(4-(2-(2-(2-((2-(2,6-dioxopiperidin-3-yl)-1,3-dioxoisoindolin-4-yl)oxy)ethoxy)ethoxy)ethoxy)phenoxy)-2-hydroxy-2-methylpropanamide (**13c**)

Compound **13c** was prepared using a similar method for the synthesis of **13a** (29%). 1H NMR (500 MHz, Chloroform-*d*), δ 8.99 (d, *J* = 2.1 Hz, 1H), 8.28 (t, *J* = 2.1 Hz, 1H), 8.11 (d, *J* = 15.2 Hz, 1H), 7.69 (ddd, *J* = 8.6, 2.1, 0.8 Hz, 1H), 7.63 (dd, *J* = 8.5, 7.3 Hz, 1H), 7.53 (dd, *J* = 8.5, 1.2 Hz, 1H), 7.42 (dt, *J* = 7.4, 0.8 Hz, 1H), 7.26–7.21 (m, 2H), 6.84–6.74 (m, 4H), 4.86 (dd, *J* = 12.4, 5.3 Hz, 1H), 4.35 (t, *J* = 9.0 Hz, 1H), 4.32 (ddd, *J* = 6.8, 3.5, 1.8 Hz, 2H), 4.05 (dd, *J* = 5.7, 4.0 Hz, 2H), 3.94 (ddd, *J* = 6.1, 3.7, 1.8 Hz, 2H), 3.88 (dd, *J* = 9.1, 2.6 Hz, 1H), 3.85–3.74 (m, 4H), 3.73 (dd, *J* = 5.8, 3.2 Hz, 2H), 3.68 (s, 1H), 2.90–2.61 (m, 3H), 2.11–2.00 (m, 1H), 1.56 (s, 3H). ^13^C NMR (126 MHz, Chloroform-*d*), δ 172.83, 171.10, 168.13, 167.06, 165.57, 156.46, 153.93, 151.97, 136.55, 134.81, 133.73, 129.50, 119.46, 119.41, 118.81, 117.25, 116.17, 115.90, 115.75, 98.97, 75.83, 73.25, 71.19, 70.85, 69.79, 69.36, 69.23, 68.10, 49.07, 29.74, 22.98, 22.58; HR-MS (EI) calculated for (M+) 826.1347; found 826.1346.

##### *N*-(4-Cyano-3-iodophenyl)-3-(4-((5-((2-(2,6-dioxopiperidin-3-yl)-1,3-dioxoisoindolin-4-yl)oxy)pentyl)oxy)phenoxy)-2-hydroxy-2-methylpropanamide (**13d**)

Compound **13d** was prepared using a similar method for the synthesis of **13a** (31%). 1H NMR (500 MHz, Chloroform-*d*), δ 8.23 (d, *J* = 2.1 Hz, 1H), 7.98 (s, 1H), 7.71–7.62 (m, 2H), 7.61–7.50 (m, 1H), 7.44 (dd, *J* = 7.3, 2.0 Hz, 2H), 7.19 (d, *J* = 8.6 Hz, 2H), 6.82–6.68 (m, 4H), 4.93 (dd, *J* = 12.0, 5.2 Hz, 1H), 4.22–4.14 (m, 2H), 4.11 (q, *J* = 7.1 Hz, 1H), 3.99 (d, *J* = 9.8 Hz, 1H), 3.91 (q, *J* = 6.2 Hz, 2H), 2.81–2.39 (m, 4H), 2.03 (d, *J* = 1.9 Hz, 2H), 1.97–1.89 (m, 2H), 1.86–1.80 (m, 2H), 1.69 (s, 3H). ^13^C NMR (126 MHz, Chloroform-*d*), δ 172.15, 170.84, 168.01, 167.09, 165.41, 156.66, 153.89, 152.14, 136.59, 134.29, 133.84, 132.06, 121.12, 118.92, 118.31, 117.15, 115.93, 115.41, 101.70, 81.53, 74.04, 69.25, 68.24, 49.11, 29.75, 28.96, 28.68, 22.66, 22.56, 19.40; HR-MS (EI) calculated for (M+) 780.1292; found 780.1298.

### 3.2. Biology

#### 3.2.1. Cell Culture and Treatment

Human embryonic kidney 293T (HEK 293T) cells and 22Rv1 cells were maintained in Dulbecco’s modified Eagle medium (DMEM, Hyclone™, SH30243.01) supplemented with 10% fetal bovine serum (FBS, Hyclone™, SH30084.03), 100 units/mL penicillin, and 100 μg/mL streptomycin (Hyclone™, SV30010). LNCaP cells and DU-145 cells were maintained in Roswell Park Memorial Institute 1640 medium (RPMI1640, Hyclone™, SH30027.01) supplemented with 10% fetal bovine serum (FBS, Hyclone™, SH30084.03), 100 units/mL penicillin, and 100 μg/mL streptomycin (Hyclone™, SV30010). All the cells were cultured in a humidified atmosphere of 5% CO_2_ and 95% air at 37 °C. All the compounds were stored in DMSO at -20 °C with a concentration of 10 mM. When treating cells, a compound was diluted in DMEM and RPMI1640 according to the concentration to use for treatment. After removing the cell medium, the compound was replaced with the diluted medium and cultured according to the desired incubation time.

#### 3.2.2. Transfection

Calcium-phosphate transfection (calcium chloride, 2 × HEPES Buffered Saline (HBS)) methods were performed for the overexpression of AR in HEK293T cells. Plasmid DNA in distilled water was mixed with 2M calcium chloride. The mixture was mixed using a vortex mixer and mixed with 2 × HBS. The final mixture was incubated at RT for 10 min and then carefully treated on the prepared HEK293T cells. HEK293T cells were incubated for 12 h in an CO_2_ incubator to stabilize and overexpression was sufficiently achieved, then used for experiments. AR plasmid (pIRESneoFLAGhAR Cat.89116) was purchased from Addgene.

#### 3.2.3. Immunoblotting

After the treatment was completed according to the experimental conditions, the cells were lightly chilled with cold PBS, separated from the cell culture plate, and centrifuged at 1200 rpm for 3 min. The pellet was sampled with 2 × sample buffer (1M TrisHCl pH 6.8, 50% glycerol, 10% SDS, 2-mercaptoethanol, 1% bromophenol blue) and then boiled at 100 °C for 10 min. After that, electrophoresis was performed with sodium dodecyl sulfate polyacrylamide gel electrophoresis (SDS-PAGE) suitable for the kDa of the protein to be detected, then transferred to a PVDF membrane activated with EtOH. Blocking was performed for a specific binding reaction between the protein of the PVDF membrane and the antibody. Blocking was performed at 4 °C overnight in a 5% bovine serum albumin (BSA, RMbio, Missoula, MT, USA) solution. The BSA solution was discarded, and the membrane was washed with PBST (PBS, 0.5% Tween-20). The specific binding reaction between the membrane protein and the antibody was performed at 4 °C overnight and washed with PBST. Finally, horse radish peroxidase (HRP)-conjugated secondary antibodies (Cell Signaling Technology, Danvers, MA, USA) were diluted in PBST and reacted for two hours at room temperature (RT). Photographing was performed using an HRP substrate (Luminata Forte (Millipore, Burlington, MA, USA)). For immunoblotting, anti-Flag rabbit antibody (Cell Signaling Technology, 14793), anti-AR rabbit antibody (Cell signaling Technology, 5153), anti-GST antibody (Abcam, Cambridge, UK), and anti-actin mouse antibody (Santacruz, D0419, Dallas, TX, USA) were used. The ImageJ program was used for intensity comparison and the analysis of all the proteins.

#### 3.2.4. RT-PCR

The mRNA levels of AR, PSA, and TMPRSS2 were analyzed by qRT-PCR. Cellular total RNA was isolated using an RNA isolation and preparation kit (GeneAll, Lisbon, Portugal, Hybrid-R 100 prep 305–101) according to the instructions provided by the manufacturers. The concentration and purity of the isolated total RNA were measured with a spectrophotometer. A total of 1000 ng of total RNA was synthesized to cDNA. Synthesis was performed as indicated in the protocol of the cDNA synthesis kit (Enzynomix, TOPsript RT DryMIX (dT 18 plus) RT200). Synthesized cDNA was subjected to qRT-PCR with SYBR Green Mix using the CFX connect real-time PCR system. The mRNA level was analyzed using AR (androgen receptor) primer, PSA (prostate-specific antigen) primer, and TMPRSS2 (transmembrane protease serine subtype2) primer. All values were normalized to the β-actin mRNA level as a control. The primer sequence of Table 1. was used as the primer used in all RT-PCR.

#### 3.2.5. Transferase-Mediated Deoxyuridine Triphosphate (dUTP)-Digoxigenin Nick End Labeling (TUNEL) Assay

The apoptotic effects of **13b** in the DU-145 and LNCaP cell lines were determined using the transferase-mediated deoxyuridine triphosphate (dUTP)-digoxigenin nick end labeling assay. Briefly, DU-145 and LNCaP cells were seeded on poly-L-lysine pre-coated 24-well cover glasses at 1 × 10^5^ cells/well. Following treatment with vehicle or **13b**, the cells were fixed and subjected to TUNEL staining using a commercial kit (In Situ Direct DNA Fragmentation (TUNEL) Assay Kit; abcam66108) in accordance with the manufacturer’s instructions. Apoptotic cells were detected as localized bright red cells (positive cells) in DIC images using a Leica DM IL microscope.

### 3.3. Molecular Modeling

#### Docking Studies

The in silico docking of **5a** with the 3D coordinates of the crystal structure of the androgen receptor ligand-binding domain W741L mutant complex with (*R*)-bicalutamide (AR, PDB code: 1Z95) was accomplished using the AutoDock 4.2 program downloaded from the Molecular Graphics Laboratory of the Scripps Research Institute. The AutoDock program was chosen because it uses a genetic algorithm to generate the poses of the ligand inside a known or predicted binding site, utilizing the Lamarckian version of the genetic algorithm where the changes in conformations adopted by molecules after in situ optimization are used as subsequent poses for the offspring. In the docking experiments carried out, water was removed from the 3D X-ray coordinates while Gasteiger charges were placed on the X-ray structures of the active site of AR along with **5a** using tools from the AutoDock suite. A grid box centered on the AR binding domain with definitions of 11.466, 18.150, and 32.019 points and a 0.37 Å spacing was chosen for the ligand docking experiments. The docking parameters consisted of setting the population size to 150, the number of generations to 27,000, and the number of evaluations to 25,000,000, while the number of docking runs was set to 50 with a cutoff of 1 Å for the root mean square tolerance for the grouping of each docking run. The docking model of androgen receptor ligand-binding domain W741L mutant complex with compound **5a** is depicted in Figure 5; the rendering of the picture was generated using Chimera 1.10 (UCSF Chimera, San Francisco, CA, USA).

## 4. Conclusions

In conclusion, we developed a series of novel PROTACs to induce the targeted degradation of AR as the potential therapeutics for the treatment of CRPC. We designed, synthesized, and biologically evaluated novel AR antagonists based on the structure of bicalutamide, which has been used clinically as an anti-androgen agent (Scheme 1). By using a novel bicalutamide analog **5a** identified as a potent AR antagonist in the cellular experiments, we prepared novel PROTACs through the conjugation of **5a**, an AR-targeting warhead, with thalidomide through a biologically compatible ethylene glycol linkage. Several novel PROTACs that we synthesized were investigated with regard to their abilities to induce targeted AR degradation. In particular, PROTAC **13b** was shown to significantly induce targeted AR degradation in a dose- and time-dependent manner. Further biological studies, such as antiproliferative activity experiments and in vivo xenograft experiments using PROTAC compounds such as **13b**, remain to be conducted.

## Figures and Tables

**Table 1:** Primer sequences for RT-PCR.

Gene	Forward Primer Sequence	Reverse Primer Sequence
AR	5′-CAGTGGATGGGCTGAAAAAT-3′	5′-GGAGCTTGGTGAGCTGGTAG-3′
PSA	5′-ACGCTGGACAGGGGGCAAAAG-3′	5′-GGGCAGGGCACATGGTTCACT-3′
TMPRSS2	5′-CAGGAGTGTACGGGAATGTGATGGT-3′	5′-GATTAGCCGTCTGCCCTCATTTGT-3′
β-actin	5′-AGTTGCGTTACACCCTTTCTTG-3′	5′-GCTGTCACCTTCACCGTTCC-3′

## Data Availability

Data is contained within the article.

## Supplementary Materials

The following are available online, Figure S1. Molecular docking model of 5a (pink) with projection of 7a (sky blue) in androgen receptor (PDB Code: 1Z95), which was visualized using Chimera 1.10 (UC13bSF Chimera).; Scheme S1. The synthesis of Pomalidomide and Derivatives.
