# Supplementary material for: Chemical Degradation of Androgen Receptor (AR) Using Bicalutamide Analog–Thalidomide PROTACs
Source: Molecules. 2021 Apr 26;26(9):2525. doi: 10.3390/molecules26092525 (PMC8123623; doi:10.3390/molecules26092525)
Supplement: Supplementary file 1 [file molecules-26-02525-s001.zip › molecules-1134725-supplementary(1).pdf]

Chemical Degradation of Androgen Receptor (AR) Using Bicalutamide Analog-Thalidomide PROTACs

Ga Yeong Kim[1,†], Chae Won Song[2,†], Yo-Sep Yang[1], Na-Rae Lee[1], Hyung-Seok Yoo[1], Seung Hwan Son[1], Soo Jin Lee[1], Jong Seon Park[1], Jong Kil Lee[1], Kyung-Soo Inn[1,2,*], Nam-Jung Kim[1,2,*]

1 College of Pharmacy, Kyung Hee University, 26 Kyungheedae-ro, Dongdaemun-gu, Seoul, 02447, Republic of Korea

2 Department of Life and Nanopharmaceutical Sciences, Graduate School, Kyung Hee University, 26, Kyungheedae-ro, Dongdaemun-gu, Seoul, 02447, Republic of Korea

Scheme S1. The synthesis of Pomalidomide and Derivatives.

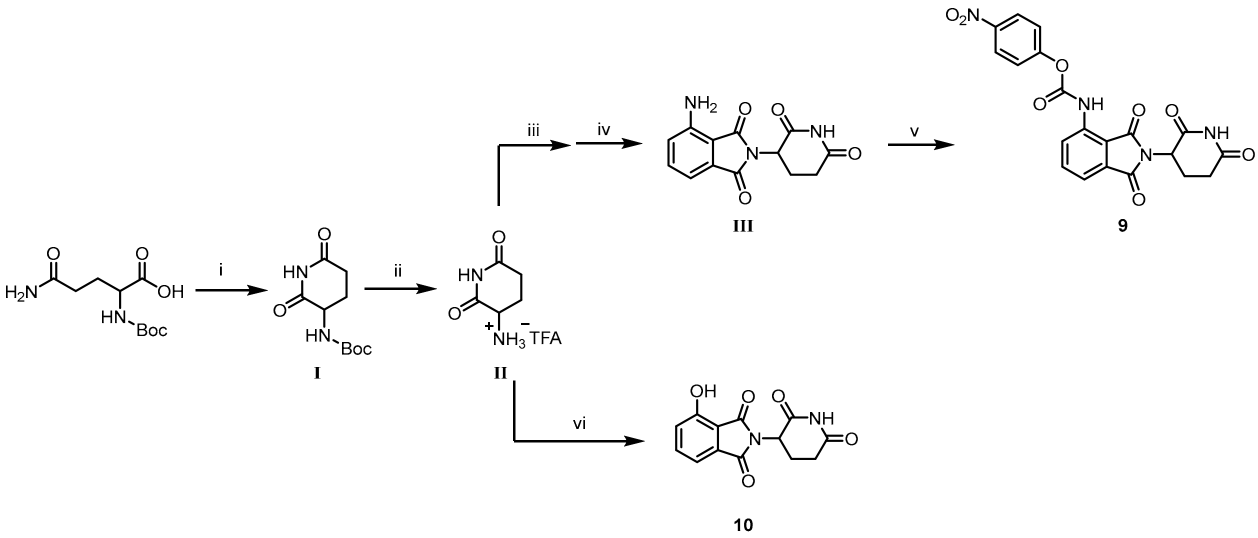

Scheme S1. Synthesis of compound **9** and **10**. Reagents and conditions: (i) DCC, NHS, DMF, 80 °**C**, 99%; (ii) TFA, CH_2Cl_2, 25 °C, 99%; (iii) 4-nitroisobenzofuran-1,3-dione, NaOAc, AcOH, reflux, 79%; (iv) Pd/C, H_2, DMF, 25 °C, 93%; (v) 4-nitrophenyl chloroformate, THF, reflux, 81%; (vi) 4-hydroxyisobenzofuran-1,3-dione, KOAc, AcOH, reflux, 51%.

Compound **9** and **10** was synthesis commenced from the well-documented pomalidomide and derivatives. Intermediate **1** was synthesized from commercially available (tert-butoxycarbonyl) Glutamine by cyclization in DCC condition [25]. Introduction of phthalimide ring used 4-nitroisobenzofuran-1,3-dione and 4-hydroxyisobenzofuran-1,3-dione as reagents were obtained **III** and **10** individually after amine deprotection [26,27]. Compound **9** was obtained substitution of amine to carbamate [28].

Figure S1. Molecular docking model of **5a** (pink) with projection of **7a** (sky blue) in androgen receptor (PDB Code: 1Z95), which was visualized using Chimera 1.10 (UCSF Chimera).

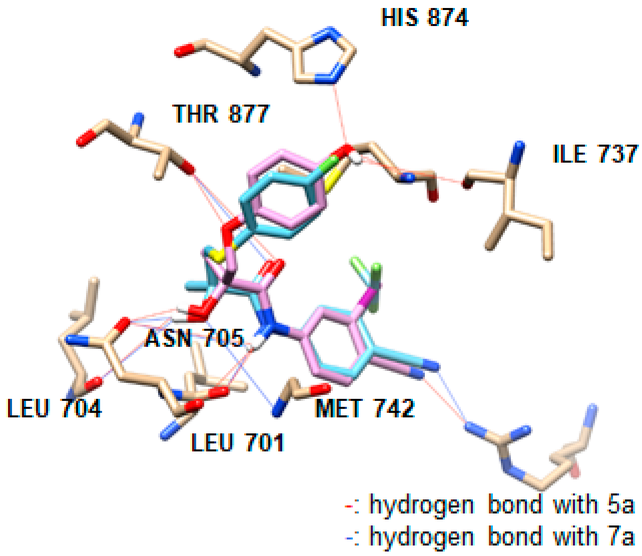

The docking model was confirmed that unlike substituent F of compound **7a** which also contained bicalutamide, the phenol part of other compound **5a** acted as acceptors of hydrogen bonds with His874 and Ile737 to form a more stable complex with the androgen receptor.
